# Deciphering disparities in childhood stunting in an underdeveloped state of India: an investigation applying the unconditional quantile regression method

**DOI:** 10.1186/s12889-020-09559-8

**Published:** 2020-10-16

**Authors:** Saswata Ghosh, Santosh Kumar Sharma, Debarshi Bhattacharya

**Affiliations:** 1 Demography and Population Health Expert, Centre for Health Policy (CHP), Asian Development Research Institute (ADRI), Patna, 800013 India; 2 Institute of Development Studies Kolkata (IDSK), Kolkata, India; 3 Centre for Health Policy (CHP), Asian Development Research Institute (ADRI), Patna, 800013 India; 4 Lead- State Health Systems, Bill & Melinda Gates Foundation, New Delhi, India

**Keywords:** Childhood stunting, Unconditional quantile regression, Counterfactual decompositions, Bihar, India

## Abstract

**Background:**

Unacceptably high rate of childhood stunting for decades remained a puzzle in the eastern Indian state of Bihar. Despite various programmatic interventions, nearly half of the under-five children (numerically about 10 million) are still stunted in this resource-constrained state.

**Data and methods:**

Using four successive rounds of National Family Health Survey (NFHS) data spread over more than two decades and by employing unconditional quantile regressions and counterfactual decomposition (QR-CD), the present study aims to assess effects of various endowments as well as returns to those endowments in disparities in childhood stunting over the period.

**Results:**

The results show that although the child’s height-for-age Z-scores (HAZ) disparity largely accounted for differing levels of endowments during the earlier decades, in the later periods, inadequate access to the benefits from various development programmes was also found responsible for HAZ disparities. Moreover, effects of endowments and their returns varied across quantiles. We argue that apart from equalizing endowments, ensuring adequate access to different nutrition-centric programmes is essential to lessen the burden of childhood stunting.

**Conclusion:**

The state must focus on intersectoral convergence of different schemes in the form of state nutrition mission, and, strengthen nutrition-centric policy processes and their political underpinnings to harness better dividend.

## Background

Child undernutrition in India has remained a priority among academicians and policy makers. Despite significant economic growth during the past two decades, prevalence of childhood stunting has dropped only by 27% (about 14 points) [1]. UNICEF (2013) has observed that India alone contributed 38% of the stunted children in the world in 2011 [2], while Headey [2013] estimated that the number of undernourished children in India was higher than that in all of Africa [3]. Jose et al. emphasized that despite a moderate decline in child undernutrition during the past decade, a large and graded socio-economic disparity in child undernutrition continues to persist [4]. A systematic review on the prevalence of child undernutrition in India has also concluded that the burden of child undernutrition is still unacceptably high in India and there is an urgent need to understand the risk factors in greater details [5]. It is needless to mention that a rapid reduction of child undernutrition in India is imperative to lessen the global burden of child malnutrition.

Majority of the studies carried out in India and other developing countries have demonstrated that an array of household, individual, and contextual factors have significant bearing on childhood undernutrition [6, 7]. Higher consumption expenditure in household lowers the risk of child malnutrition [6, 7], while economic gradients contribute in maintaining the vicious cycle of poverty and malnutrition [8–10]. Lack of household sanitation [11, 12], low body-mass index (BMI) among mothers [13–15], less parental education [16], lack of bargaining power of women within households [17] significantly and negatively affect the child’s anthropometric outcomes in low- and middle-income countries.

Recent studies found that women’s BMI, education, child’s adequate diet, household assets and sanitation, age at marriage, antenatal care and household size are strong and significant predictors of childhood anthropometric outcomes and explain much of the variations across the districts in India [18, 19]. Jose et al. have noted that about 83% of high stunting prevalence (higher than the national average) districts belong to the eight states located in the north-central, western, and eastern region [4]. Thus, effects of endowments (or covariate per se) were found to be significant; however, they vary across the space and nature of endowments.

On the other hand, attempts have been made by some studies to document disparities in returns to endowments (or strength of association per se) and their different dimensions, which potentially influence child nutritional outcomes. For example, these studies have tried to document how quality of the governance, institutional strength in implementing public policies, reach of public services, bargaining power of the communities, and macro-level politico-economic context etc. could influence health and nutritional outcomes. In the Indian context, disparity in institutional performance (measured in terms of the quality of public services such as health, education and public distribution system) was observed between northern and north-central states, and southern states [20–22]. Harriss and Kohli investigated the influence of inter-state political and institutional factors on child undernutrition and differentiated between the politics of “clientelism” and “programmatic” politics [23]. They argued that such political spectrum could impinge on worse and better child anthropometric outcomes respectively. Significant gaps in implementation regarding the nature, coverage and quality of Integrated Child Development Services (ICDS) were found by various researchers in different states [24–27]. Using conditional quantile regression model, Mukhopadhyay (2013) found that while the presence of government facilities was able to make a positive difference in the upliftment of nutritional status for the relatively better-off children in India, it had not benefitted the worse affected children much [28].

The majority of literature reviewed above have either tried to identify some of the key observable characteristics (or covariates or endowments) that help in explaining variation in child anthropometric outcomes or have emphasized that the differential strength of relationship (or coefficients or returns to endowments) might also influence childhood nutritional outcomes. Instances of quantifying the influence of socio-demographic, economic and ecological variables, individually or at the aggregate level (covariate effects) and the contribution of the strength of relationship (or coefficient effects) together were limited in the South Asian context [29, 30]. In the present context, covariate effects can be defined as the differences in nutrition outcomes across periods explained by the differences in observed covariates. On the other hand, differences explained by differing strengths of relationships between covariates and outcomes, in other words the “returns” to specific endowments, can be termed coefficient effects. To find out differentials in child undernutrition in Nepal and Bangladesh, Srinivasan et al. have highlighted that rural-urban disparities in child nutrition are primarily attributable to the difference in the levels of critical endowments such as household affluence, maternal as well as spouse’s education, while differences in the strength of association (or returns to endowments) between determinants and nutrition outcomes are relatively small in magnitude [29]. However, studies conducted in India found that large disparities in child nutritional outcomes across states are modestly explained by the differences in critical endowments, while returns to endowments or implementation of nutrition-relevant programmes are crucially related in explaining such disparity [30].

During the past two decades, India and its states have witnessed substantial changes in endowments (covariates) and have also experienced enormous policy changes (coefficients) which could have direct or indirect bearing on child nutritional outcomes. Apart from the expanding scope and coverage of ICDS, many states have also come up with many state-specific schemes and emphasized multisectoral nutrition intervention. For example, Maharashtra, Madhya Pradesh and Karnataka have implemented State Nutrition Missions and placed special emphasis on nutrition surveillance, district planning, and district-level monitoring with the goal of reducing undernutrition to a desirable extent.

The present study intends to find out the changing relative contribution of different covariates and coefficients resulting in disparities in childhood stunting in different intervals between 1992-93 and 2015-16 in the state of Bihar. The state of Bihar, located in the eastern part of India is a resource-constrained state, having the highest prevalence of childhood stunting in India for several past decades. The proportion of childhood stunting has declined by 21% (or by 13 percentage-points) during the last 20 years – implying an annual average decline of just 1 % [1]. Numerically, about 10 million children in Bihar are stunted. Notably, Bihar alone contributes around 15% of stunted children in India. More importantly, out of 100 districts in India, where prevalence of stunting is the highest, one-quarter belongs to Bihar. It was estimated that malnutrition (maternal and child malnutrition together) continued to be the largest risk factor driving maximum death and disability since 1990s [31].

Changes in the basic socio-demographic and economic indicators during the last two decades are given in Table 1. To note, the state of Bihar has undergone territorial changes following Bihar Reorganization Act (2000) (Government of India, 2000) and a separate state of Jharkhand was created from the districts of south Bihar.

**Table 1:** Some important demographic and health indicators of Bihar in 1992-93 and 2015-16

Demographic and Health Indicators	1992-93	2015-16
Sex ratio (female/1000 male)	956^a^	1062^c^
% population aged 6+ that is literate	44.6^a^	66.9^c^
% female population aged 6+ that is literate	28.6^a^	57.0^c^
Child (0-6 years) sex ratio	944^a^	939^c^
Infant mortality rate	89.2^a^	48.2^c^
Total fertility rate	3.25^a^	3.40^c^
% mothers who had at least 3 ANC for last birth	30.7^a^	14.4^c^
% skilled attendance at delivery	19.0^a^	70.0^c^
% institutional delivery	13.0^a^	63.8^c^
Head count poverty ratio	61%^b^	34%^b^
Singulate Mean age at marriage	18.0^a^	19.5^d^

Sources: ^a^NFHS 1 (1992-93); ^b^World Bank 2016; ^c^ NFHS 4 (2015-16); ^d^IIPS 2016

The strength of the present study is as follows. First, although the under-five children of Bihar are highly vulnerable to stunting compared to those in the other states of India for long, hardly any comprehensive study has been carried out in Bihar covering almost two and half decades to understand the changing relative contribution of endowments and returns to endowments resulting in disparities in childhood stunting. Second, the study applied an advanced econometric tool, namely, unconditional quantile regression-based counterfactual decomposition (QR-CD) method, which allows a more nuanced approach to disentangle the effects of endowments (or covariates) and returns to endowments (or coefficients) and thus contributes to the existing literature on childhood stunting in India.

The study would also like to enquire whether the changing contribution of endowments and their returns are different at the lower quantile of height-for-age Z-score (HAZ) distribution, where the likelihood of prevalence of severe stunting is more than the middle and higher ends of the HAZ distribution. Such insights would be of utmost value in a policy atmosphere where targeting the most vulnerable is considered imperative. The primary hypothesis is that the period-wise changes across the HAZ distribution arises from covariate, rather than coefficient effects. Disparities at the lower end of the HAZ distribution are of specific attention to the present study. A secondary hypothesis is that, there are important differences across the quantiles in terms of relative contributions of endowments and returns to endowments in period-wise changes, even if an endowment or its return dominates.

## Materials and methods

### Data and variables

Data for this study was drawn from the four successive rounds of National Family Health Survey (NFHS), an Indian variant of Demographic and Health Surveys (DHS) (www.dhsprogram.com), which were carried out between 1992-93 and 2015-2016 by the IIPS, Mumbai; ORC Macro; Macro International Inc. and ICF [1, 32–34]. It is imperative to note that although the state of Bihar was reorganized in 2000, data was culled out for the second round (1998-99) for the districts representing present-day Bihar using district codes to make it comparable with the third round (2005-06). During 1992-93, in undivided Bihar, the survey collected information of 3575 children born during the 4 years preceding the survey. During 1998-99, 2005-06 and 2015-16 information of 2948; 2320 and 3679 children were collected respectively. During 1998-99 and 2005-06 information was collected for the children born during 3 years preceding the survey, while such duration was for 5 years during 2015-16. For this reason, the study has been restricted to children of age group 0 – 36 months in order to compare childhood stunting over the four rounds of NFHS. It may be noted that the present study intends to compare changes of covariate and coefficient effects between two successive rounds such as between NFHS 1 and NFHS 2; NFHS 2 and NFHS 3; and; NFHS 3 and NFHS 4, and not over the rounds, for instance, between NFHS 1 and NFHS 4.

Stunting has been defined as height-for-age Z-scores (HAZ) less than minus two standard deviation of the WHO International Reference Standard [35]. It is universally considered as a standard indicator of child undernutrition and health status as it reflects chronic undernutrition caused by long-term deprivation. A child’s height-for-age is a measure of their height, relative to a healthy standard population of the same sex and the same age-in-months. Height-for-age is measured using Z-scores, meaning that it is expressed as a difference between the height of the observed child and the average height of healthy children, scaled by the standard deviation of child height in the healthy population. A child with a height-for-age Z-score (HAZ) of zero would be as tall as the average child in the healthy reference population; a child with a negative height-for-age Z-score is shorter than the average child in the healthy reference population. The formula for calculating the HAZ score is.

$$ \mathrm{HAZ}=\frac{\mathrm{Observed}\ \mathrm{height}-\mathrm{average}\ \mathrm{height}}{\mathrm{Standard}\ \mathrm{Deviation}\ \left(\mathrm{SD}\right).} $$

Complete information on HAZ score was available for 1821; 1627; 1188; and 2184 children of age 0-36 months in the four respective rounds. HAZ has been used as outcome variable in all the regression models. The study has included the current age of the child (in months), square of the age, sex of the child (male, female), size of the child at birth (more than average, average, small) as a proxy for birth weight, initiation of early breastfeeding (no, yes), and number of siblings as child characteristics. Receipt of any services from ICDS during 12 months preceding the survey was included while comparing NFHS 3 and 4 because such information was available only in these rounds. Maternal characteristics comprises age of the mother at first birth (in years), maternal education (in completed years), work status (working, not working), degree of media exposure (additive index of three binary variables – reading newspaper, watching television, listening to radio at least once a week). Institutional delivery (no, yes) was considered as a proxy of contact with health personnel by the mother. Maternal height and maternal BMI, and anaemia (no, mild, moderate and severe) were included for analyses of second, third and fourth rounds of NFHS because such information were not collected in the first round. Similarly, normalized factor scores of variables indicating household decision making, freedom of movement etc. were incorporated as maternal level variable in second, third and fourth rounds of NFHS^1^.

Household wealth index, religious category (Hindu, Muslims/others), membership to social group (scheduled castes (SC), scheduled tribes (ST), Others) were incorporated as household level variables. One may note here that the first round of NFHS did not collect data on ‘other backward castes’ (OBCs) and thus categorised them as ‘Others’. Household wealth index as calculated by DHS is based on possession of household durable assets, availability of safe drinking water and sanitation, and landholding. For construction of index, the variables were first broken into sets of dichotomous variables and indicator weights were then assigned using principal component analyses (PCA) as suggested by Filmer and Pritchett [36]. In addition to the variables representing child, maternal and household characteristics, place of residence (rural/urban) was also included in the regression models.

### Statistical analysis

To assess the differentials in HAZ scores over the study period, first, the distribution of the HAZ scores of Bihar’s children was estimated separately in each survey period using kernel smoothing techniques and period-wise differentials were computed at each quantile to provide raw difference in HAZ scores across the distribution.

The present study was intended to decompose the period-wise differences in child’s HAZ scores in covariate effect, i.e. the differences in HAZ scores arising out of the differences in levels of characteristics or composition of the children in the survey-period; and the coefficient effect, i.e. the differences in HAZ scores caused by the differences in the returns to those characteristics or structure, across the entire HAZ distribution. It is worth noting that most of the earlier studies have largely modelled the nutrition outcomes (such as HAZ scores) at the mean level by using ordinary least square (OLS), or the prevalence of stunting, underweight or wasting by using logit or probit regression approaches. These approaches have limitation on the following grounds. First, changes in the covariates and the effect of covariates are constrained to be same along the entire distribution of outcome variable (HAZ, in this case) in these models. Second, decompositions based on OLS would apply only to the period-wise mean differences in HAZ scores; however, not to other distributional characteristics, such as quantiles.

Quantile regression (QR) method, developed by Koenker and Bassett, allows effects of covariates to vary across the entire distribution of continuous response variable [37]. Limitation of this model is that it estimates only the conditional quantile effects of changes in covariates. In this study, we intended to estimate the effect of policy intervention, for instance, mother’s BMI in a population of individuals with different characteristics (i.e. unconditional effects) rather than its association for some sub-groups with explicit values of mother’s BMI (i.e. conditional effects). Unconditional recentred influence function quantile regression developed by Firpo et al. to assess the unconditional quantile effects of changes in covariates was employed in the present study [38]. The method consists of employing a regression of a transformation – the recentered influence function (RIF) – of the dependent variable (Y) on the explanatory variables (X). Advantage of this method is that it allows estimating the contribution of each explanatory variable for the components of the HAZ decomposition and thus extends the Blinder-Oaxaca (BO) decomposition to distributional statistics other than the mean [39]. The details of the differences between conditional and unconditional quantile methods are given in the Appendix Note. The rationale behind application of such quantile regression based counterfactual decomposition (QR-CD) approach would be strengthened if there are important differences across the HAZ distribution in the relative contributions of covariate and coefficient effects to period-wise changes.

To estimate the unconditional quantile regression, first we have derived the RIF of the response variable (HAZ score, in our case). The RIF for the *τ*_*th*_ quantile is given by the following expression:

1 $$ RIF\left(Y,{q}_{\tau}\right)={q}_{\tau }+\frac{\tau -I(Y)\le {q}_{\tau }}{f_y\left({q}_r\right)} $$

Where *f*_*Y*(*qτ*)_ is the marginal density function of Y at the point q_*τ*_ estimated by kernel methods; q_*τ*_ is the sample quantile; I (Y ≤ q_*τ*_) is an indicator function, which indicates whether the value of the response variable is below q_*τ*_. RIF offers a linear approximation to a non-linear functional (ν(Y)) (such as median) of the Y distribution and thus permits calculating partial effects for every covariate [38]. Firpo et al. have also shown that by estimating OLS of the new transformed response variable on the covariates (X), the RIF quantile regression may be implemented [38]. In case of this study, considering two periods (t_1_ and t_2_), RIF regressions for HAZ score in both periods are estimated as:

2 $$ E\ \left[ RIF\ \left({Y}_{i\epsilon g};{q}_{\tau }\ \right)\ |\ {X}_{i\epsilon g}\ \right]={X}_{i,g}\ {\beta}_{\tau, g}\ g={t}_1,{t}_2 $$

Coefficients *β*_*τ, g*_ represents the approximate marginal effects of the predictor variables on the HAZ quantile *q*_*τ*_ for children age 0-35 months in periods *g = t*_*1*_*, t*_*2*_.

Once we estimate the parameter β_τ, g_ for each year in the sample, OB decomposition is applied using RIF unconditional quantile estimates for any given quantile by following equation -

$$ \hat{q_{\tau }}\left({HAZ}_{t\_2}\right)-\hat{q_{\tau }}\left({HAZ}_{t\_1}\right)=\left[\overline{X_{t\_2}}\left(\hat{\beta_C}\right.-\left.\hat{\beta_{t\_2}}\right)+\hat{R^{Coeff}}\right]+\left[\left(\overline{X_{t\_1}}\hat{\beta_{t\_1}}-\overline{X_{t\_2.}}\hat{\beta_C}\right)+\hat{R^{Cov}}\right] $$

where t_2_ is the final year and t_1_ is the initial year. In our application, we set up the initial years as 1992-93, 1998-99, and 2005-06 and the final years as 1998-99, 2005-06, and 2015-16 respectively. As typical in OB decomposition, the term $\hat{q_{\tau }}\left({HAZ}_{t\_2}\right)-\hat{q_{\tau }}\left({HAZ}_{t_{\_1}}\right)$ represents the raw differences in *t*_2_ and *t*_1_ HAZ scores at the τ^th^ quantile and X represents the covariate averages. The term $\overline{X_{t\_2}}\left(\hat{\beta_C}\right.-\left.\hat{\beta_R}\right)$ refers to the coefficient effect and $\left(\overline{X_{t\_1}}\hat{\beta_{t\_1}}-\overline{X_{t\_2}}\hat{\beta_C}\right)$ represents the differences between *t*_2_ and *t*_1_ scores, which are attributed to the differences in characteristics of the endowments and thus refers as the covariate effect. $\hat{R^{Coeff}}$ and $\hat{R^{Cov}}$ are error terms while estimating coefficient and covariate effects.

Although the current research started with the reduced form of conceptual framework of the UNICEF [40], a further refinement of covariate set was required, since decomposition of observed HAZ differences into covariate and coefficient effects require well-specified regressions models which should include key relevant covariates [30]. The final regression models include the covariates representing child, maternal, household and spatial characteristics as mentioned in the preceding section.

To note, we have tried our best to minimize endogeneity problems in accordance with the previous literature [29, 30], though some form of endogeneity bias may persist and can lead to difficulties in parameter interpretation. However, as O’Donnell et al. noted, the aim of the CD exercise is not solely to identify causal relationship, but to explain variations in child’s HAZ and resolve the relative values of covariate and coefficient effects [41]. One should cautiously interpret the coefficients of variables that are potentially endogenous; however, the decomposition itself remains valid.

## Results

### Descriptive statistics

Table 2 reveals percentile of HAZ scores adjusted by kernel smoothing for the four rounds of NFHS. HAZ scores of first and second rounds of NFHS are not strictly comparable because of territorial changes as mentioned earlier. The HAZ values of Bihar were also compared with the HAZ values of overall India. Without loss of generality, one can note that absolute increase in overall HAZ scores was the highest between the second and third rounds of NFHS (i.e. between 1998-99 and 2005-06) followed by the third and fourth rounds i.e. between 2005-06 and 2015-16. Child’s HAZ scores largely remained at the same level between 1992-93 and 1998-99. Absolute increase of child’s HAZ scores was remarkable for the bottom quantiles between 1998-99 and 2005-06 nationally and in Bihar, in particular. In Bihar, there was even decline of HAZ scores at the top quantile. However, between 2005-06 and 2015-16, absolute increase in the HAZ scores was observed at the top quantile nationally as well as in Bihar. In other words, nutritionally better-off children gained more compared to the severely stunted during the last decade.

**Table 2:** Percentile of HAZ score in NFHS 1, NFHS 2, NFHS 3 and NFHS 4 in Bihar and India

Bihar	NFHS 1	NFHS 2	NFHS 3	NFHS 4	Absolute increase btw NFHS 1 and NFHS 2	Absolute increase btw NFHS 2 and NFHS 3	Absolute increase btw NFHS 3 and NFHS 4
10	−4.92	− 4.89	− 4.03	−3.74	0.03	0.86	0.29
25	−3.85	−3.75	−2.95	− 2.81	0.10	0.80	0.14
50	−2.49	− 2.46	−1.93	− 1.80	0.03	0.54	0.13
75	− 1.09	− 1.08	−0.84	−0.61	0.01	0.24	0.23
90	0.12	0.36	0.19	0.65	0.24	−0.17	0.46
Overall	−2.36	−2.28	−1.89	−1.63	0.07	0.40	0.26
**India**	**NFHS 1**	**NFHS 2**	**NFHS 3**	**NFHS 4**	**Absolute increase btw NFHS 1 and NFHS 2**	**Absolute increase btw NFHS 2 and NFHS 3**	**Absolute increase btw NFHS 3 and NFHS 4**
10	−4.26	−4.19	−3.72	−3.49	0.07	0.47	0.24
25	−3.16	− 3.10	−2.70	−2.48	0.06	0.40	0.22
50	−2.00	−1.96	−1.63	−1.43	0.04	0.33	0.20
75	−0.85	−0.82	−0.51	−0.27	0.03	0.31	0.24
90	0.32	0.31	0.64	0.99	−0.01	0.33	0.35
Overall	−1.94	−1.91	−1.55	−1.30	0.03	0.36	0.25

Source: Computed by the authors from unit-level data of NFHS rounds

Table 3 depicts socio-demographic and economic characteristics of the samples in four rounds of NFHS. It has been observed that initiation of early breastfeeding (within 1 h of birth) has improved dramatically – more than 14-times – between 2005-06 and 2015-16. Although number of siblings of the index child has declined in the recent past, it still indicates that fertility in the state is high. Notably, benefit received from ICDS services increased by more than 7-fold between 2005-06 and 2015-16. Similar is the case for institutional delivery of mothers. Mother’s age at first child has increased by nearly 2 years during the study period. BMI of mothers has improved between 2005-06 and 2015-16, while the rate of decline of anaemia was substantial between 1998-99 and 2005-06 compared to 2005-06 and 2015-16. Mother’s educational level has improved marginally in all the rounds. Although workforce participation rate among mothers remained consistent at around 20% during 1992-93 and 2005-06, it has declined by half between 2005-06 and 2015-16. Degree of media exposure was found to have increased marginally over the years.

**Table 3:** Sample Characteristics of child age 0-35 months according to background characteristics in Bihar

	NFHS 1	N	NFHS 2	N	NFHS 3	N	NFHS 4	N
Child HAZ [*mean (SD)]*	−2.36 (0.05)	1821	−2.29 (0.05)	1627	−1.89 (0.05)	1188	−1.63 (0.04)	2184
*Age of Child in Month [mean (SD)]*	16.3(9.6)	1821	16.4(10.6)	1627	17.5(10.1)	1188	17.7(10.1)	2184
*Age2 [mean (SD)]*	357.2(342.7)	1821	378.5(376.0)	1627	405.5(367)	1188	414.7(373.7)	2184
***Child Sex***								
*Male*	49.5	902	52.1	847	53.5	635	51.8	1131
Female	50.5	919	47.9	779	46.6	553	48.2	1053
**Birth Size**								
Normal	71.9	1309	69.5	1130	47.2	561	69.34	1514
Average and above	11.0	200	14.5	236	31.5	374	17.42	380
*Small*	17.1	312	16.1	261	21.3	253	13.24	289
**Early Breastfeeding**								
*No*	98.2	1766	95.6	1406	97.4	1146	63.4	1151
*Yes*	1.8	30	4.4	65	2.6	31	36.6	665
*No. of Siblings [mean (SD)]*	1.88(1.7)	1821	1.96(1.8)	1627	2.06(1.9)	1188	1.62(1.4)	2184
**Benefitted ICDS services**								
No	–		–		92.1	1094	39.0	853
*Yes*	–		–		7.9	94	61.0	1331
***Mother’s Characteristics***								
Institutional Delivery								
No	87.6	1594	84.9	1382	77.98	924	30.8	673
Yes	12.4	227	15.1	245	22.02	262	69.2	1511
**Age of mother at first birth** [mean (SD)]	18.55(2.9)	1821	18.28(2.8)	1627	18.51(3.0)	1188	20.42(3.1)	2184
**BMI of mother [mean (SD)]**	**–**		19.34(2.5)	1627	19.30(2.6)	1188	20.06(3.2)	2184
***Mother’s anaemia***								
*No*	–		71.1	1156	73.1	849	65.84	1438
*Yes*	–		28.9	470	26.9	313	34.16	746
*Mother’s height in cm [mean (SD)]*	–		149.6(5.4)	1627	150.0(5.4)	1188	149.5(5.8)	2184
**Mother’s Education (mean)**	2.02(4.0)	1821	2.01(3.8)	1627	2.93(4.5)	1188	3.98(4.9)	2184
***Working mother***								
*No*	78.1	1422	80.6	1306	79.9	950	89.2	1964
*Yes*	21.9	399	19.7	321	20.1	238	10.2	220
Media exposure (range)	0.47 (0-3)	1821	0.43 (0-3)	1627	0.60 (0-3)	1627	1.31(0-3)	2184
**Religion**								
Hindu	78.2	1424	82.4	1340	81.6	969	79.2	1731
Muslim/Others	21.8	397	17.6	287	18.4	219	20.8	453
**Caste**								
SC	9.3	169	23.7	386	19.6	233	19.9	433
ST	8.5	156	1.3	21	0.9	10	3.7	81
Others	82.2	1496	75.0	1220	79.6	945	76.4	1670
**Wealth index**								
Poorest	20.5	373	18.2	369	31.7	376	55.5	1217
Poorer	25.9	471	20.4	414	33.3	396	24.8	539
Middle	16.9	308	23.3	472	16.6	197	11.6	251
Richer	13.8	252	23.2	471	12.5	149	6.3	137
Richest	22.9	417	14.9	303	5.8	69	1.8	39
**Place of residence**								
Urban	13.1	238	6.2	101	11.1	132	10.8	236
Rural	86.9	1583	93.8	1526	88.9	1056	89.2	1948
Total	100.0	1821	100.0	1627	100.0	1188	100.0	2184

Source: Computed by the authors from unit-level data of NFHS rounds; SD: standard deviation

Majority of the respondents in the sample was Hindu and non-SC/ST, including OBCs. It is surprising to find out that proportion of economically marginalized households in the sample has increased from 1998-99 to 2015-16, in spite of the state’s higher economic growth during these periods, particularly after 2005-06 [42]. Being the least urbanised state of the country (among the major states), overwhelming proportion of the sample belong to the rural areas of Bihar.

### Unconditional RIF quantile regression results

The estimates derived from unconditional RIF quantile regressions (QR) separately for all the survey periods were shown in Tables 4 and 5. It has been observed that child age has negative and significant influence with child’s HAZ scores across quantiles. If one moves from the lower tail to the upper tail of the distribution, this effect increases. It indicates that the children, who have started with better nutritional status tend to lose more as they grow older through faltering. Although such observation holds for the second and third rounds of the survey, the said observation confirms up to 75% quantile for the first and fourth rounds. Girls were found to have significantly better HAZ outcomes compared to boys across quantiles; however, strength of association varies across quantile and period of survey. Child’s size at birth (proxy for birth weight) was found to have varying association with HAZ scores across quantiles during first two rounds; in third and fourth rounds, size of the children at birth did not have any significant effect on HAZ scores. Early initiation of breastfeeding was found to have positive and significant effect on HAZ scores in the first round, while such effect weakened during the last three rounds. Higher sibling size has negative significant influence on child’s HAZ scores, particularly among those belonging to the lower quantiles in the third and fourth rounds of the survey. Receipt of any benefit from ICDS was found to be negatively associated with child’s HAZ scores and such effect increases when we go from the lower tail to the higher tail of the HAZ distribution in the last round of the survey.

**Table 4:** Unconditional Re-centred Influence Function (RIF) quantile regression results for NFHS 1 (1992-93) and NFHS 2 (1998-99) in Bihar

	NFHS 1					NFHS 2				
	10	25	50	75	90	10	25	50	75	90
Age of Child	−0.073*** (0.006)	−0.103*** (0.0078)	− 0.171*** (0.006)	− 0.173*** (0.007)	− 0.086*** (0.012)	0.025 (0.062)	− 0.104* (0.055)	− 0.237*** (0.060)	− 0.303*** (0.085)	− 0.419** (0.195)
Age^2^	0.001*** (0.0002)	0.001*** (0.0002)	0.003*** (0.0002)	0.003*** (0.0002)	0.001*** (0.0003)	−0.003 (0.002)	0.000 (0.002)	0.004** (0.002)	0.006*** (0.002)	0.010* (0.005)
*Female*	0.234*** (0.035)	0.306*** (0.040)	0.344*** (0.032)	0.367*** (0.034)	0.578*** (0.054)	0.442 (0.289)	0.625** (0.305)	1.089*** (0.348)	0.412 (0.422)	0.480 (0.842)
**Birth Size**										
*Normal*										
*Average and above*	0.054 (0.054)	−0.102 (0.065)	0.077 (0.050)	0.485*** (0.060)	0.332*** (0.095)	0.218 (0.404)	0.580 (0.500)	0.833* (0.451)	0.944 (0.652)	2.332 (1.472)
*Small*	−0.185*** (0.049)	− 0.003 (0.054)	0.066 (0.044)	0.002 (0.047)	0.088 0.074)	−0.505 (0.518)	0.416 (0.404)	0.476 (0.465)	1.127* (0.642)	−0.291 (1.111)
Early Breastfeeding (Yes)	0.820*** (0.035)	1.063*** (0.095)	0.447*** (0.127)	0.422*** (0.142)	−0.242 (0.192)	−1.281 (0.927)	−1.137 (0.787)	−0.117 (0.783)	− 0.064 (1.228)	−2.467** (1.091)
No. of Siblings	−0.003 (0.010)	0.007 (0.012)	−0.017* (0.009)	− 0.013 (0.010)	0.036** (0.016)	− 0.089 (0.126)	− 0.014 (0.095)	0.109 (0.100)	0.146 (0.116)	0.158 (0.211)
**Mother’s Characteristics**										
*Institutional Delivery (yes)*	0.398*** (0.054)	0.888*** (0.066)	0.398*** (0.061)	0.135** (0.065)	−0.192** (0.094)	0.472 (0.579)	1.680*** (0.611)	2.231** (0.956)	1.943 (1.298)	4.823 (3.050)
Age of mother at first birth	−0.021*** (0.005)	0.005 (0.007)	−0.004 (0.006)	0.020*** (0.007)	0.051*** (0.011)	0.061 (0.063)	0.018 (0.063)	0.045 (0.063)	0.000 (0.072)	−0.006 (0.159)
BMI of mother						0.000 (0.0004)	0.000 (0.0004)	0.001*** (0.0003)	0.001 (0.0008)	0.003 (0.002)
**Mother’s anaemia**										
Yes	–	–	–	–	–	0.258 (0.368)	−0.274 (0.373)	− 0.998*** (0.384)	−1.256*** (0.469)	−2.049** (0.903)
Mother’s height						0.006** (0.003)	0.003 (0.003)	0.005* (0.003)	0.006* (0.004)	−0.003 (0.008)
Mother’s Education	0.019*** (0.005)	0.003 (0.007)	0.025*** (0.006)	0.030*** (0.007)	0.025** (0.010)	−0.003 (0.084)	−0.047 (0.097)	− 0.100 (0.089)	−0.187** (0.075)	− 0.213 (0.201)
Working mother	−0.228*** (0.048)	− 0.551*** (0.054)	− 0.371*** (0.040)	− 0.240*** (0.041)	−0.357*** (0.062)	− 1.16** (0.568)	− 1.52*** (0.553)	− 0.868 (1.089)	−0.331 (1.132)	0.354 (3.189)
Empowerment						−0.096 (0.141)	0.019 (0.128)	−0.252** (0.103)	−0.193 (0.129)	− 0.316 (0.239)
Media exposure	−0.016 (0.030)	0.011 (0.033)	0.157*** (0.028)	0.172*** (0.034)	0.241*** (0.053)	0.020 (0.281)	−0.247 (0.484)	−0.341 (0.440)	− 0.394 (0.458)	−1.629* (0.927)
**Religion**										
Hindu										
Muslim/Others	−0.115*** (0.045)	−0.141*** (0.052)	− 0.073* (0.042)	−0.115*** (0.043)	0.075 (0.068)	−0.468 (0.585)	0.481 (0.501)	0.912 (0.619)	0.802 (0.765)	2.375 (1.774)
**Caste**										
SC										
ST	0.480*** (0.085)	1.018*** (0.098)	0.360*** (0.077)	0.179** (0.082)	−0.435*** (0.138)	0.126 (1.215)	−1.027 (0.831)	−0.097 (0.830)	0.325 (1.132)	0.369 (2.086)
Others	0.355*** (0.070)	0.765*** (0.077)	0.168*** (0.057)	−0.095 (0.063)	−0.533*** (0.138)	0.030 (0.361)	−0.637* (0.353)	− 0.100 (0.371)	−0.249 (0.447)	0.407 (0.871)
**Wealth index**	−0.004 (0.018)	0.137*** (0.020)	0.086*** (0.017)	0.053*** (0.018)	0.193*** (0.030)	−0.028 (0.210)	−0.192 (0.199)	− 0.053 (0.189)	−0.008 (0.241)	− 0.086 (0.475)
**Place of residence**										
Urban										
Rural	0.241*** (0.061)	0.523*** (0.068)	0.027 (0.055)	−0.282*** (0.065)	−0.131 (0.099)	−1.262* (0.723)	0.304 0.984)	1.695* (0.923)	0.144 (1.139)	−1.302 (2.430)
Constant	−4.321*** (0.145)	−3.87*** (0.166)	−1.037*** (0.138)	0.336** (0.162)	0.426 (0.278)	−11.677*** (4.289)	−6.495 (4.362)	−12.044*** (4.633)	− 10.286* (5.978)	2.286 (12.925)
R square	0.0423	0.0938	0.1361	0.1318	0.0456	0.211	0.247	0.3207	0.2267	0.174
Adj. R square	0.0414	0.0929	0.1352	0.131	0.0446	0.127	0.167	0.2482	0.1443	0.0859

***p < 0.001; **p < 0.01; *p < 0.05; Source: Computed by the authors from unit-level data of NFHS 1 and NFHS 2; Values in parenthesis are standard errors

**Table 5:** Unconditional Re-centred Influence Function (RIF) quantile regression results for NFHS 3 (2005-06) and NFHS 4 (2015-16) in Bihar

	NFHS 3					NFHS 4				
	10	25	50	75	90	10	25	50	75	90
Age of Child	−0.021 (0.038)	− 0.100*** (0.024)	− 0.148*** (0.022)	− 0.153*** (0.032)	− 0.215*** (0.040)	−0.095*** (0.025)	− 0.106*** (0.020)	− 0.171*** (0.020)	− 0.197*** (.030)	− 0.124*** (0.037)
Age2	−0.001 (0.001)	0.001* (0.0007)	0.002*** (0.0006)	0.002** (.0008)	0.004*** (0.001)	0.002*** (0.0007)	0.002*** (0.0006)	0.003*** (0.0005)	0.004*** (0.0008)	0.002** (0.0009)
*Female*	0.172 (0.183)	−0.02 (0.126)	0.024 (0.114)	0.139 (0.150)	0.177 (0.172)	0.355*** (0.127)	0.237** (0.103)	0.167* (0.100)	0.065 (0.140)	0.193 (0.174)
**Birth Size**										
*Normal*										
*Average and above*	0.064 (0.209)	0.037 (0.147)	0.089 (0.132)	0.276 (0.175)	−0.007 (0.193)	0.109 (0.150)	0.142 (0.163)	0.189 (0.135)	0.237 (0.201)	0.226 (0.259)
*Small*	−0.227 (0.248)	−0.102 (0.166)	− 0.187 (0.149)	− 0.109 (0.195)	− 0.106 (0.222)	− 0.256 (0.218)	− 0.245 (0.106)	−0.203 (0.149)	0.166 (0.215)	0.432 (0.307)
Early Breastfeeding (yes)	0.538 (0.484)	0.715** (0.336)	0.326 (0.371)	0.449 (.434)	0.102 (0.484)	0.105 (0.127)	−0.017 (0.041)	0.049 (0.104)	0.038 (0.147)	0.047 (0.186)
No. of Siblings	−0.112** (0.055)	−0.075** (0.035)	− 0.052* (0.031)	−0.013 (.041)	0.012 (0.047)	−0.117** (0.054)	− 0.039 (0.106)	0.025 (0.038)	− 0.018 (0.051)	−0.036 (0.071)
Benefitted ICDS services (yes)	−0.187 (0.388)	− 0.150 (0.251)	0.156 (0.205)	0.158 (.275)	0.362 (0.362)	−0.069 (0.128)	−0.245** (0.106)	− 0.259** (0.107)	− 0.183 (0.150)	− 0.619*** (0.199)
**Mother’s Characteristics**										
*Institutional Delivery*	− 0.206 (0.261)	−0.224 (0.171)	− 0.085 (.1490)	− 0.016 (.204)	− 0.235 (0.230)	0.090 (0.148)	0.224* (0.122)	−0.098 (0.114)	− 0.235 (0.168)	− 0.041 (0.210)
Age of mother at first birth	− 0.022 (0.035)	− 0.009 (0.024)	0.009 (0.021)	− 0.017 (.029)	0.004 (0.035)	0.000 (0.019)	0.000 (0.017)	0.005 (0.016)	0.009 (0.021)	0.066** (0.028)
BMI of mother	0.001* (0.0004)	0.001*** (0.0002)	0.001*** (0.0002)	0.001*** (.0003)	0.001*** (0.0003)	0.001*** (0.0002)	0.000*** (0.000)	0.001*** (0.0001)	0.000 (0.0002)	0.000 (0.0003)
**Mother’s anaemia**										
Yes	−0.275 (0.205)	−0.292** (0.143)	− 0.120 (0.130)	− 0.171 (.170)	−0.088 (0.196)	− 0.200 (0.130)	0.007 (0.109)	0.118 (0.106)	−0.047 (0.146)	− 0.027 (0.178)
Mother’s height	0.004** (0.001)	0.006*** (0.001)	0.005*** (0.001)	0.005*** (.001)	0.008*** (0.001)	0.006*** (0.001)	0.004*** (0.001)	0.006*** (0.001)	0.006*** (0.001)	0.005*** (0.001)
Mother’s Education	0.017 (0.028)	0.019 (0.019)	0.025 (0.019)	0.045 (.028)	0.014 (0.033)	0.025 (0.015)	0.014 (0.013)	0.015 (0.013)	−0.033* (0.018)	0.001 (0.024)
Working mother	0.283 (0.281)	0.199 (0.183)	−0.125 (0.152)	− 0.084 (.196)	0.38* (0.206)	−0.241 (0.198)	− 0.316* (0.162)	− 0.051 (0.159)	− 0.210 (0.233)	0.231 (0.253)
Empowerment	− 0.097 (0.067)	− 0.073 (0.048)	0.011 (0.046)	0.059 (.061)	0.011 (0.070)	0.003 (0.047)	0.007 (0.109)	0.036 (0.037)	0.037 (0.053)	0.081 (0.064)
media exposure	0.090 (0.215)	0.148 (0.147)	0.131 (0.131)	−0.037 (.167)	−0.162 (0.176)	− 0.033 (0.043)	−0.019 (0.034)	− 0.005 (0.033)	0.034 (0.046)	0.001 (0.060)
**Religion**										
Hindu										
Muslim/Others	0.187 (0.245)	−0.015 (0.170)	0.108 (0.157)	0.286 (.216)	−0.290 (0.209)	0.035 (0.162)	−0.099 (0.145)	−0.118 (0.141)	− 0.407** (0.193)	−0.409 (0.253)
**Caste**										
SC										
ST	1.489*** (0.494)	0.427 (0.898)	0.149 (0.707)	−0.094 (1.048)	−0.429 (0.411)	0.249 (0.170)	0.023 (0.130)	0.087 (0.120)	−0.065 (0.174)	−0.029 (0.221)
Others	0.069 (0.275)	0.259 (0.186)	0.319** (0.160)	0.529*** (.179)	0.309* (0.181)	0.305 (0.212)	0.146 (0.183)	0.369** (0.182)	0.160 (0.258)	−0.041 (0.329)
**Wealth index**	0.000** (0.000)	0.000** (0.000)	0.000** (0.000)	0.000** (0.000)	0.000 (0.000)	0.000 (0.000)	0.000 (0.000)	0.000** (0.000)	0.000** (0.000)	0.000 (0.000)
**Place of residence**										
Urban										
Rural	0.207 (0.197)	0.023 (0.136)	0.006 (0.138)	0.265 (.1940)	0.085 (0.224)	−0.177 (0.194)	− 0.089 (0.170)	− 0.132 (0.170)	− 0.173 (0.250)	0.101 (0.297)
Constant	−9.868*** (3.174)	−12.311*** (1.948)	−9.598*** (1.762)	−9.526*** (2.390)	−11.783*** (2.836)	− 12.537*** (2.092)	−8.243*** (1.664)	− 9.426*** (1.656)	−7.241*** (1.983)	−6.973*** (2.338)
R square	0.090	0.171	0.209	0.176	0.124	0.061	0.094	0.177	0.115	0.062
Adj. R square	0.072	0.155	0.193	0.159	0.107	0.049	0.083	0.167	0.104	0.050

***p < 0.001; **p < 0.01; *p < 0.05; Source: Computed by the authors from unit-level data of NFHS 3 and NFHS 4; Values in parenthesis are standard errors

Institutional delivery of mother, which is an important indicator for contact with health personnel, has positive and significant influence on child’s HAZ scores across quantiles, particularly at the lower and middle quantile in varying degree except during the third round of the survey. Significant positive effect of higher age of mother’s first birth on child’s HAZ outcomes was found in the higher quantiles during the first and the latest rounds of the survey, but not in the other rounds. Notably, significant positive influence of maternal education on child’s HAZ scores decreased with rounds.

Working mothers are significantly more likely to have children with lower HAZ scores compared to their non-working counterparts across quantiles during the first round of the survey; however, such association holds only in lower quantiles in the second and fourth rounds. Mother’s exposure to any mass media was found to have positive significant influence in the middle and upper quantiles of HAZ scores in the first round, though it weakened in other rounds. Maternal height and BMI both have small but significant influence in enhancing child’s HAZ scores across quantiles; and such association strengthened in the last two rounds of survey. Degree of maternal empowerment was found to have positive significant effect on child’s HAZ scores during the second round of the survey; however, such relationship weakened during the last two rounds.

Differentials with respect to religious affiliation were found in child’s HAZ scores during the first round of the survey; however, the relationship weakened thereafter – indicating that differences in religion is no more a significant factor in explaining disparities in childhood stunting. However, relationship between childhood stunting and caste affiliation is not straightforward – significant differentials were observed in the first and the third rounds of the survey; however, not in the second and fourth rounds. Significant positive influence of household affluence on child’s HAZ outcomes was found during the first and third round of the survey and observation suggests that the effect is higher among those belonging to higher quantiles. The results also revealed that rural-urban differentials in child’s HAZ outcomes diminished over the period in Bihar.

### Quantile regression Oaxaca Blinder counterfactual decomposition (QR-CD)

The estimated QR-CD results at the aggregate level of child, maternal, household and spatial characteristics were presented in the Tables 6, 7 and 8, while a detailed breakdown of contribution of these characteristics were given in the Appendix Tables A1-A3.

**Table 6:** Oaxaca Blinder decomposition of HAZ scores of NFHS 1 and NFHS 2 in Bihar

		10		25		50		75		90
NFHS 1 HAZ score		−4.935***		−3.870***		−2.511***		−1.160***		0.098
NFHS 2 HAZ score		−4.812***		−3.659***		−2.339***		−0.930***		0.455***
Observed Raw gap in HAZ scores		**−0.124**		−0.211***		−0.172**		−0.230**		−0.358***
Covariate effect		−0.059*		−0.050		−0.108**		−0.114***		−0.106
(% contribution)		47.7		23.7		62.9		49.6		29.7
Coefficient Effect		−0.065		−0.161		−0.064		−0.116		−0.251*
(%contribution)		52.3		76.3		37.1		50.4		70.3
	**Covariate effect**					**Coefficient effect**				
	**10**	**25**	**50**	**75**	**90**	**10**	**25**	**50**	**75**	**90**
Aggregate effect	−0.059*	− 0.050	− 0.108**	− 0.114***	− 0.106	−0.065	− 0.161*	−0.064	− 0.116	−0.251*
Child Characteristics	−0.193***	−0.260***	− 0.261***	−0.255***	− 0.088	0.389	0.195	0.134	−0.818**	0.342
(%)	142.1	270.8	112.7	95.6	36.5	− 949.8	−263.9	−192.1	534.7	− 559.9
Mother’s Characteristics	− 0.032**	− 0.037**	− 0.033*	− 0.010	0.021	− 0.204	−0.240	− 0.116	0.117	− 0.067
(%)	23.6	38.4	14.1	3.6	−8.8	498.0	324.5	166.3	−76.4	110.1
Household’s Characteristics	0.105*	0.235***	0.064	−0.021	−0.186	− 0.102	− 0.059	− 0.162	− 0.021	0.126
(%)	− 77.5	−244.6	− 27.4	7.8	77.2	247.9	79.6	231.6	13.6	−206.4
Spatial Characteristics	−0.016	− 0.034**	− 0.002	0.019	0.012	0.419	−0.110	− 0.184	− 0.081	−0.631
(%)	11.9	35.8	0.9	−7.1	−4.8	− 1022.3	148.0	262.3	52.9	1033.8
Constant						−0.543	0.139	0.258	0.649	0.169
Residuals	0.077	0.047	0.124	0.153	0.135	−0.024	−0.087	0.006	0.037	−0.19
Total	−0.136	−0.096	− 0.232**	−0.267***	− 0.241	−0.041	− 0.074	−0.07	− 0.153	−0.061

***p < 0.001; **p < 0.01; *p < 0.05; Source: Computed by the authors from unit-level data of NFHS 1 and NFHS 2

**Table 7:** Oaxaca Blinder decomposition of HAZ scores of NFHS 2 and NFHS 3 in Bihar

		10		25		50		75		90
NFHS 2 HAZ score		−5.036***		−4.043***		−2.908***		−1.294***		0.474
NFHS 3 HAZ score		−4.064***		−3.027***		−1.983***		−0.854***		0.153
Observed Raw gap in HAZ scores		−0.972***		−1.016***		−0.925***		−0.440*		**0.321**
Covariate effect		−0.384		−0.112		0.393		0.68		0.991
(% contribution)		39.5		11		−42.5		−154.5		309.3
Coefficient Effect		−0.588		−0.905**		−1.317***		−1.121**		−0.671
(%contribution)		60.5		89		142.5		254.5		−209.3
	**Covariate effect**					**Coefficient effect**				
	**10**	**25**	**50**	**75**	**90**	**10**	**25**	**50**	**75**	**90**
Aggregate effect	−0.384	−0.112	0.393	0.680	0.991	−0.588	−0.905**	−1.317***	−1.121**	− 0.671
Child Characteristics	0.034	−0.032	−0.207	− 0.415	−1.136**	− 1.702***	− 1.667***	− 0.775***	− 0.897***	−1.777***
(%)	− 2.2	2.2	90.1	− 418.8	397.3	−136.4	−150.9	181.0	− 1008.0	− 265.7
Mother’s Characteristics	− 1.941*	− 1.409	− 0.349	0.630	1.811	9.36***	22.151***	6.782***	6.046***	13.241***
(%)	123.4	98.8	151.9	636.3	−633.4	750.1	2004.7	− 1584.5	6792.7	1979.2
Household’s Characteristics	0.114	−0.300	− 0.151	− 0.058	− 0.352	− 0.102	−1.856***	−0.184	− 0.373**	0.020
(%)	−7.2	21.0	65.8	−58.1	123.0	−8.1	−167.9	43.0	−419.0	2.9
Spatial Characteristics	0.220	0.314	0.478	−0.058	− 0.610	0.214	0.163	0.192	0.284	0.232
(%)	−14.0	−22.0	−207.8	−59.1	213.1	17.2	14.8	−44.9	318.8	34.7
Constant						−6.524*	−17.687***	−6.443***	−4.971***	− 11.046***
Residuals	1.189	1.315	0.623	0.581	1.278	−1.836	−2.009	−0.889	−1.209	− 1.34
Total	−1.573	− 1.427	− 0.23	0.099	− 0.286	1.248***	1.105***	−0.428***	0.089	0.669***

***p < 0.001; **p < 0.01; *p < 0.05; Source: Computed by the authors from unit-level data of NFHS 2 and NFHS 3

**Table 8:** Oaxaca Blinder decomposition of HAZ scores of NFHS 3 and NFHS 4 in Bihar

		10		25		50		75		90
NFHS 2 HAZ score		−4.041***		−3.016***		−1.975***		−0.843***		0.166**
NFHS 3 HAZ score		− 3.593***		−2.654***		−1.664***		−0.463		0.699***
Observed Raw gap in HAZ scores		−0.448***		−0.361***		−0.311***		−0.380***		−0.533***
Covariate effect		−0.206		0.061		0.168		0.261		0.026
(% contribution)		45.9		−17.0		−53.9		−68.7***		−4.9
Coefficient Effect		−0.243		−0.423**		−0.479***		−0.642		−0.559**
(%contribution)		54.1		117.0		153.9		168.7		104.9
	**Covariate effect**					**Coefficient effect**				
	**10**	**25**	**50**	**75**	**90**	**10**	**25**	**50**	**75**	**90**
Aggregate effect	−0.206	0.061	0.168	0.266	0.029	−0.243	− 0.423**	− 0.479***	− 0.646***	−0.562
Child Characteristics	0.424	0.220	−0.412	1.088***	0.940**	0.429*	0.849***	3.344***	0.650**	1.349***
(%)	140.5	−21.2	16.1	25.4	107.5	2873.0	256.0	247.2	−52.1	−449.1
Mother’s Characteristics	0.456*	−0.463	−1.319	1.041***	−0.160	6.123***	−2.277	−18.339***	10.574***	2.242
(%)	151.2	44.6	51.5	24.3	−18.3	41,039.2	−687.0	− 1355.7	− 847.8	− 746.3
Household’s Characteristics	−0.582	− 0.789	− 0.849	2.145***	− 0.200	− 0.935***	−0.958**	−1.711*	0.733*	−0.016
(%)	−192.9	76.1	33.2	50.1	−22.9	− 6268.0	− 289.0	−126.5	−58.7	5.3
Spatial Characteristics	0.004	−0.005	0.020	0.008	0.014	0.076	−0.246	0.906**	0.163	0.816***
(%)	1.2	0.5	−0.8	0.2	1.6	510.8	−74.3	66.9	−13.1	−271.5
Constant						−5.678**	2.965	17.154**	−13.367***	−4.692
Residuals	−0.507	1.099	2.728	−4.015	−0.846	− 0.258	−0.754	−1.831	0.601	−0.262
Total	0.302	−1.037	−2.560	4.281***	0.874	0.015	0.332	1.353	−1.247***	−0.300

***p < 0.001; **p < 0.01; *p < 0.05; Source: Computed by the authors from unit-level data of NFHS 3 and NFHS 4

Before interpreting the results, it should be kept in mind that the negative sign of the observed raw gap in HAZ scores between two successive periods reflects the fact that raw HAZ scores of the later period were lower than the previous period in all quantiles, except at the highest quantile between the second and third rounds. Further, it must be recognized that the direction of effect of the contribution of characteristics as shown in the Tables 6, 7 and 8 – negative figures implies a contribution to *increase* in the disparity in HAZ scores over time, while positive figures shows a contribution to *diminish* it over the periods. A careful look at these tables reveals certain patterns of covariate effects and coefficient effects across quantiles and over the periods.

It may be observed that between the periods 1992-93 and 1998-99 covariate (or endowments) effects contributed significantly to enhance disparities in child HAZ outcomes, at the 10th, 50th and 75th quantiles, while coefficient (returns to endowments) effects dominated over covariate effects in enhancing disparities in child’s HAZ outcomes at 90th quantile (see Table 6). Lower panel of the Table 6 suggests that child endowments alone contributed 36.5% at 90th quantile to 270.8% at 25th quantile in explaining disparities in child’s HAZ outcomes. Effect of mother’s characteristics (or mother’s endowments) in explaining such disparities was found to be relatively small and varies between − 8.8% at 90th quantile to 38.4% at 25th quantile, while effects of household characteristics reduced covariate effects, particularly at 10th and 25th quantiles.

Notwithstanding, the directions of covariate and coefficient effects reversed significantly between the periods 1998-99 and 2005-06 as well as between 2005-06 and 2015-16 (see Tables 7 and 8). During both the periods, coefficient effects (or returns to endowments) significantly surpassed covariate effects (or endowments) in most quantiles except the bottom quantile. Between 1998-99 and 2005-06, coefficient effects enhanced disparities in child’s HAZ outcomes by 89–254.5% between 25th and 75th quantiles (Table 7), while such effects vary between 117 and 168.7% between the same quantile between 2005-06 and 2015-16 (Table 8). Additionally, between the said periods, coefficient effects enhanced disparities in child’s HAZ outcomes even at the 90th quantile. The lower panels of the Tables 7 and 8 revealed that between 1998-99 and 2015-16, coefficient effects of child characteristics significantly increased disparities across quantiles, while the said effects of mother’s characteristics have tried to reduce it except at 25th and 50th quantiles. Further, coefficient effects of the household attributes have tried to increase disparities in HAZ outcomes significantly at 25th and 75th quantiles between 1998- 99 and 2005-06, and at 10th and 50th quantiles between 2005-06 and 2015-16. Additionally, during the last period, positive and significant covariate effects were observed at the higher tails of HAZ distribution.

If covariate effects and coefficient effects of different attributes are looked at in more disaggregated manner during the study period (as given in the Appendix A1-A3), it may be found that these effects vary across quantiles, periods and nature of endowment. For example, delivery in institutions was found to have significant effect in enhancing disparities, particularly at lower tails of the HAZ distribution between 1992-93 and 1998-99 *(*Appendix Table A1). Coefficient effects of mother’s height and BMI, and media exposure have tried to reduce disparities across quantiles between 1998-99 and 2005-06 *(*Appendix Table A2). During the same period, covariate effect of institutional delivery has contributed significantly in increasing disparities. Between 2005-06 and 2015-16, both covariate and coefficient effects of the receipt of ICDS services were found to be significantly associated with the reduction of HAZ disparities among children *(*Appendix Table A3). The same table also reveals that between 2005-06 and 2015-16, both covariate and coefficient effects have contributed in enhancing disparities in childhood stunting among ST children, who are placed at the lower and middle HAZ quantiles compared to the children from other caste groups.

## Discussion

The QR-CD method provides specific insight into the drivers of disparities across child’s HAZ distribution. The understanding of factors resulting in disparities in the lower quantiles of HAZ scores would be useful in designing interventions aimed at the vulnerable households with children of the highest levels of stunting. In order to assess the contribution of the ‘returns’ of various interventions in reducing child HAZ disparities during the last two decades, such quantification of the contribution of different socio-demographic, economic and cultural determinants seemed to be imperative for the state of Bihar.

This study indicates that although between 1992-93 and 1998-99 child’s HAZ disparity at the bottom quantile of the distribution largely accounted for the differing levels of endowments, in the later periods such differences weakened statistically except for the children belonging to the socially marginalized ST community. In other words, between 1992-93 and 1998-99, at the lowest quantile, reducing disparity in childhood stunting was a matter of equalizing endowments; however, between 1998-99 and 2015-16, both unequal endowments as well as dissimilar access to the benefits of implementation of government sponsored schemes were largely responsible for childhood HAZ disparity. At the higher quantiles, particularly between 50 – 75th quantile, although unequal endowments were responsible for such disparities between 1992-93 and 1998-99, inadequate access to benefits from programme implementation was largely found accountable between 1998-99 and 2005-06 as well as between 2005-06 and 2015-16.

From the QR-CD estimates between 2005-06 and 2015-16, it is important to note that there are limited number of equalizing endowments which can have significant influence in reducing disparities in child HAZ outcomes for the bottom quantiles, though at the aggregate level, influences of endowments were statistically weak. According to the current estimates, much of the reduction of disparities at the lowest quantile can be achieved by maintaining the regularity of ICDS services, early initiation of breastfeeding, reduction in sibling size (proxy for fertility size), increasing mother’s age at first birth, mass media exposure, educational attainment, employability, and social inclusivity. Additionally, access to the programmes pertaining to initiation of early breastfeeding, securing access and reducing gender-gap in receipt of ICDS services, reduction of early childbearing, improving mother’s nutritional status, and creation of household wealth were found to be imperative to the households having the highest level of stunting. Because coefficient effects indicate all-inclusive returns to endowments, arguably, not only the *reach* of these programmes, but also ensuring *quality* of these programmes also could enhance child nutritional status. Although earlier studies have also demonstrated the influence of these characteristics in lowering stunting [43, 44], they were unable to quantify the influence of reach of various policies and programmes in reducing stunting.

In addition to the implementation of centrally sponsored schemes such as ICDS, the government of Bihar has started various programmes in the recent past which have indirect influence on the reduction of child undernutrition. Currently 18 centrally sponsored schemes and 30 state-specific nutrition-sensitive schemes are being implemented by 16 departments covering all the aspects of the findings of the present study. It would have been more meaningful and easier to monitor if all these schemes were brought under a single umbrella of a State Nutrition Mission. How a State’s Nutrition Mission can successfully reduce the menace of child undernutrition has been well-documented for the state of Maharashtra in India [45]. The key factors identified in the policy processes include the way in which the issue was framed and available evidences played a catalytic role for a political response. Forming the State Nutrition Mission was, thus a response of government structures; and system-wide capacity was combined with leadership in an innovative fashion to utilize available resources.

Nonetheless, the Draft State Plan of Action for Children 2017 proposed 11 strategies and actions for all-round development for children. These include effective implementation of schemes, programmes and laws; mapping vulnerable households and linking those households with appropriate development schemes; raising community awareness on the nutritional issues through institutional interventions; institutional strengthening through capacity building of staff; improved infrastructure and outreach; strengthening child-relevant resources and facilitating uptake of principal schemes and services etc. The state plans for action also emphasized ‘breaking the intergenerational cycle of malnutrition’ by provisioning take-home ration and ensuring safe health and hygiene practices through better outreach services, particularly in the aspirational districts. The said action plan must also accommodate the issue of intersectoral coordination in implementation of these programmes in order to harness better dividend of these schemes.

There are some limitations of the study, which need to be pointed out. First, NFHS sampling frame of 1998-99 does not allow the separation of districts from the states. However, because of the unavailability of any other comparable dataset, it was compelling to segregate districts of undivided Bihar. This may under- or over-estimate the QR-CD results to a disproportionate extent. Secondly, CD exercise can provide reliable estimates only if the primary quantile regression includes all the important factors of childhood stunting and is well-specified [29]. To note, as the choice of explanatory variables has been constrained by the coverage of NFHS, key variables considered by the previous literature are included in the present study [18, 29, 30]. However, in such situation, the issue of endogeneity cannot be entirely ruled out, though necessary tests were carried out to get rid of this. Thirdly, providing clinical interpretations of the effect size of the variables is beyond the scope of the present study. Finally, the ‘coefficient effects’ in such comparisons lump several potential effects together and are not informative about specific factors or actions [30]; thus, interpretations of coefficient effects are speculative. Nonetheless, this research helps to highlight important dimensions of child nutritional improvement during the last two and half decades for the state of Bihar.

### Conclusions and policy recommendations

Inconspicuous presence of child nutrition in the Millennium Development Goals (MDG) framework with an imperfect measure of child undernutrition (i.e. underweight) was criticised. However, the issue has gained considerable momentum in the Sustainable Development Goals (SDGs) as the ambition to ‘end hunger, achieve food security and improved nutrition and promote sustainable agriculture’ is captured in SDG 2. Further, at least 12 of the 17 Goals contain indicators, which are highly relevant to nutrition because of the fact that **w**ithout adequate and sustained investments in good nutrition, the SDGs would not be realised. The present study suggests that child undernutrition in Bihar is not just from a lack of sufficient and adequately nutritious and safe food, but from a host of intertwined factors linking social inclusivity, healthcare, women’s education and work, household wealth (including water, sanitation and hygiene), access to public distribution system (PDS) and more.

One such state-specific nutritional intervention has been implemented through JEEViKA^2^ platform in 101 blocks of 11 districts though technical support from non-governmental agencies. The said platform consists of convergence with government entitlements, nutrition education and direct livelihoods interventions such as kitchen garden, poultry, dairy cattle rearing, and food security (credit) line/fund to smooth out lean seasons (agriculture production and remittances). Initial evaluation suggests that the collectivization of healthy practices around reproductive, maternal, neonatal and child health in rural Bihar has increased significantly through this intervention [46]. It is hoped that such nutrition-sensitive programmes with improved surveillance would result in the reduction of child undernutrition in the long run. We suggest that such interventions should be scaled-up further, if proven successful. Further, the nutritional rehabilitation centres across the state must be strengthened in line with Madhya Pradesh [47]. Additionally, tightening the implementation mechanism of the PDS in the tribal areas of Bihar would be an important step. The concerned governmental agencies must ensure that tribal families are not deprived of the ration that they are entitled to because of issues like the non-possession of identity cards. To ensure better dividends from these schemes, there is a need for developing a comprehensive framework for appropriate budgeting and expenditure for these schemes and bringing convergence and greater coordination among the administrative departments [48, 49].

In addition to scaling-up proven nutrition-specific interventions in other Indian states, the state of Bihar, must focus on policy processes and their political underpinnings to reduce the risk of childhood stunting. This is of utmost importance because earlier studies have shown that strong programme leadership, political support across sectors, encouraged by personal relationships and dedication to pushing the nutrition agenda forward, and policy and programme advocacy by civil society organizations can bring about tangible outcomes in reducing disparities in childhood stunting [45].

## Data Availability

The datasets generated and/or analysed during the current study are available in the [DHS] repository, [www.dhsprogram.com]

## Appendix

### Appendix Note

**Conditional and Unconditional Quantile Regression**

Conditional quantile regression is used to assess the impact of a covariate on a quantile of the outcome conditional on specific values of other covariates. In most cases, conditional quantile regression may generate results that are often not generalizable or interpretable in a policy or population context. In contrast, the unconditional quantile regression method provides more interpretable results as it marginalizes the effect over the distributions of other covariates in the model.

The conditional quantile regression estimator by Koenker and Basset (1978) for the *τ*_*th*_ quantile is defined as

$$ {\beta}_{\tau}^{CQR}=\underset{\beta_{\mathrm{r}}\varepsilon\;{R}^p}{\min }{\varSigma}_{i=1}^n{\rho}_{\tau}\;\left( yi--{z}_i^{\hbox{'}}{\beta}_{\tau}\right) $$

where *ρ*_*τ*_ = *ui*·(*τ* − 1(*ui* < 0)) is a re-weighting function (called “check”-function) of the residuals *ui*.

Firpo et al. (2009) states that conditional quantile regression does not give the interesting effects and cannot be generalized to the population (in OLS we can always go from conditional to unconditional via the law of iterated expectations but this is not available for quantiles). This is because the τ_th_ unconditional quantile *y*_*i*_ *might* not be the same as the *τ*_*th*_ conditional quantile *y*_*i*_*|X*_*i.*_

To overcome this limitation of conditional quantile regression Firpo et al.(2009) suggest a UQR model based on the concepts of influence function (IF) and recentered influence function (RIF), as used in the robust statistics literature (Hampel et al., 1986). An IF is an analytical tool that can be used to assess the effect (or ‘influence’) of removing/adding an observation on the value of a statistic, *ν****(****F*), without having to recalculate that statistic and is defined as

$$ \mathrm{IF}\kern0.5em \left(y;\nu (F)\right)=\kern0.5em {\lim}_{\in \to 0}\left[\upnu \left(\left(1-\in \right).F+\in .\delta y\right)-\nu (F)\right]/\in, 0\le \in \le 1 $$

where *F* represents the cumulative distribution function for *Y* and δ*y* is a distribution that only puts mass at the value *y*.

An RIF is obtained by adding the statistic to its IF:

$$ \mathrm{RIF}\ \left(y;\nu \right)=\nu (F)+\mathrm{IF}\ \left(y;\nu \right) $$

One convenient feature of RIF is that its expectation is equal to that of *v*(*F*).

**Table 9:** Oaxaca Blinder decomposition of HAZ scores of NFHS 1 and NFHS 2 in Bihar

	Covariate effect					Coefficient effect				
	10	25	50	75	90	10	25	50	75	90
Aggregate effect	−0.059*	− 0.050	− 0.108**	− 0.114***	−0.106	− 0.065	−0.161*	− 0.064	− 0.116	− 0.251*
Child age in months	− 0.129***	− 0.176***	− 0.323***	− 0.304***	−0.198**	− 0.121	0.497	0.083	−1.297**	0.356
(%)	94.4	182.6	139.0	113.7	82.1	296.6	− 670.6	−118.3	845.5	− 584.9
Age2	0.032	0.029	0.104***	0.098***	0.038	0.427	−0.194	− 0.052	0.423	− 0.173
(%)	−23.4	−30.3	−44.6	−36.5	−15.7	− 1047.3	261.8	74.3	− 276.0	283.3
*Female*	0.014*	0.018**	0.022**	0.022**	0.044**	0.030	−0.088	0.043	0.001	0.040
(%)	−10.1	−18.2	−9.4	−8.1	− 18.4	−73.5	118.0	−61.0	− 0.5	− 66.2
**Birth Size**										
*Normal*										
*Average and above*	−0.002	0.003	−0.003	− 0.016**	− 0.015	− 0.034	0.008	− 0.006	0.021	0.010
(%)	1.3	−3.5	1.2	6.1	6.1	82.9	−11.1	9.2	−13.5	−16.6
*Small*	−0.003	0.000	0.001	0.000	0.002	0.037	0.027	0.009	0.002	0.113*
(%)	1.9	0.0	−0.4	0.0	−0.7	−90.3	−36.4	−12.2	−1.2	− 185.6
Early Breastfeeding (Yes)	−0.106*	− 0.134**	− 0.062	− 0.055	0.041	0.011	0.010	0.000	0.037	0.013
(%)	77.7	139.0	26.8	20.4	−17.0	− 26.3	−14.1	−0.1	−24.1	−21.1
No. of Siblings	0.000	0.000	0.000	0.000	0.000	0.040	−0.066	0.059	−0.005	− 0.019
(%)	0.00	0.01	−0.01	−0.01	0.04	−97.95	89.04	−84.49	3.08	30.62
**Mother’s Characteristics**										
*Institutional Delivery (yes)*	−0.015*	−0.032***	− 0.016*	− 0.005	0.009	0.025	−0.018	0.040	0.024	0.100*
(%)	10.9	33.7	6.9	1.9	−3.9	−61.3	24.6	−56.7	−15.3	− 163.8
Age of mother at first birth	−0.010	0.002	−0.002	0.009	0.031	−0.279	−0.278	− 0.032	0.049	− 0.151
(%)	7.2	−2.3	0.9	−3.5	−13.0	683.6	375.3	46.1	−32.2	247.6
Mother’s Education	−0.005	− 0.001	− 0.007	−0.008	− 0.009	0.025	0.041	0.018	−0.019	− 0.043
(%)	3.9	0.7	3.1	3.1	3.7	−61.7	−55.5	−25.4	12.3	71.2
Working mother	−0.002	− 0.006	− 0.004	− 0.003	−0.005	0.032	0.023	−0.077*	0.029	0.064
(%)	1.8	5.9	1.8	1.0	2.0	−77.5	−31.4	110.1	−18.7	− 105.0
Media exposure	0.000	0.000	−0.003	−0.003	− 0.006	− 0.007	−0.008	− 0.065	0.034	− 0.037
(%)	− 0.2	0.2	1.3	1.2	2.3	18.1	10.7	92.6	−22.3	60.3
**Religion**										
Hindu										
Muslim/Others	−0.007	−0.009	− 0.005	− 0.007	0.006	− 0.021	0.004	0.019	0.082	0.108
(%)	5.3	8.9	2.1	2.7	−2.6	52.4	−4.8	−26.7	− 53.7	− 177.1
**Caste**										
SC										
ST	0.021*	0.045***	0.017	0.008	−0.025	−0.005	− 0.004	− 0.007	− 0.010	0.008
(%)	−15.7	− 46.2	−7.5	−3.0	10.5	12.7	5.0	9.9	6.5	−12.5
Others	0.091*	0.192***	0.047	−0.024	−0.179*	− 0.074	− 0.046	−0.204	− 0.116	−0.020
(%)	−67.1	−199.5	−20.1	9.1	74.3	181.9	62.1	292.3	75.7	33.4
**Wealth index**	0.000	0.007	0.005	0.003	0.013	−0.001	−0.013	0.030	0.023	0.031
(%)	0.2	−6.8	−2.0	−1.0	−5.2	2.5	17.1	−43.3	−15.0	−50.5
**Place of residence**										
Urban										
Rural	−0.016	− 0.034**	− 0.002	0.019	0.012	0.419	−0.110	−0.184	− 0.081	− 0.631
(%)	11.9	35.7	0.9	−7.1	−4.8	− 1028.7	147.7	262.9	52.8	1035.0
Constant						−0.543	0.139	0.258	0.649	0.169
Residuals	0.077	0.047	0.124	0.153	0.135	−0.024	− 0.087	0.006	0.037	−0.190
Total	−0.136	−0.096	− 0.232**	−0.267***	− 0.241	− 0.041	− 0.074	−0.070	− 0.153	−0.061

****p* < 0.001; ***p* < 0.01; **p* < 0.05; Source: Computed by the authors from unit-level data of NFHS 1 and NFHS 2

**Table 10:** Oaxaca Blinder decomposition of HAZ scores of NFHS 2 and NFHS 3 in Bihar

	Covariate effect					Coefficient effect				
	10	25	50	75	90	10	25	50	75	90
Aggregate effect	−0.384	− 0.112	0.393	0.680	0.991	−0.588	− 0.905**	−1.317***	−1.121**	− 0.671
Child age in months	− 0.004	0.060	0.144	0.154	0.191	−1.256	−1.393**	− 1.570***	−1.733***	−4.301***
(%)	0.3	−4.2	−62.5	154.8	−66.8	−100.6	− 126.2	366.4	− 1956.9	− 642.6
Age2	0.131	−0.073	− 0.300	− 0.353	− 0.479	− 0.039	1.223***	0.506**	0.515**	2.071***
(%)	−8.3	5.1	130.4	− 355.5	167.5	−3.1	110.7	−118.1	581.2	309.5
*Female*	0.092	0.172**	0.174**	0.132	0.118	0.074	−0.082	−0.008	0.117**	0.154**
(%)	−5.8	−12.0	−75.8	132.6	−41.3	5.9	−7.4	1.8	132.0	23.0
**Birth Size**										
*Normal*										
*Average and above*	−0.093	−0.186	−0.338**	− 0.485**	− 0.818*	− 0.025	−0.401***	0.086**	0.139***	0.040
(%)	5.9	13.0	146.9	− 488.6	285.7	−2.0	−36.3	−20.1	156.5	5.9
*Small*	−0.006	0.015	0.070	0.146	−0.056	−0.106*	− 0.646***	0.050*	− 0.002	0.027
(%)	0.4	−1.1	−30.6	147.0	19.4	−8.5	−58.5	−11.7	−2.7	4.1
Early Breastfeeding (Yes)	−0.051	− 0.034	0.002	− 0.046	− 0.126	0.015	− 0.041	− 0.009	0.009	0.002
(%)	3.3	2.4	−1.0	−45.9	44.1	1.2	−3.7	2.1	10.7	0.2
No. of Siblings	−0.034	0.015	0.040	0.038	0.033	−0.365***	−0.327***	0.170***	0.059	0.229***
(%)	2.2	−1.0	−17.2	38.2	−11.5	−29.2	−29.6	−39.7	66.1	34.2
**Mother’s Characteristics**										
*Institutional Delivery (yes)*	−0.487	− 0.884**	−1.064***	− 0.874*	−2.486**	− 0.048	− 0.514***	− 0.042	−0.048	− 0.040
(%)	31.0	62.0	462.6	− 880.1	868.7	− 3.8	−46.5	9.9	−54.0	−5.9
Age of mother at first birth	−0.016	− 0.007	− 0.040	0.039	− 0.001	−0.773	1.568***	−0.236	− 0.359	0.293
(%)	1.0	0.5	17.3	38.9	0.3	−61.9	141.9	55.2	− 405.6	43.8
BMI of mother	0.002	−0.002	0.000	−0.007	− 0.018	1.440	5.112***	1.139***	1.469***	1.046*
(%)	−0.1	0.2	0.2	−7.0	6.3	115.3	462.8	− 265.7	1659.2	156.3
**Mother’s anaemia**										
Yes	0.048	0.343	0.614**	0.878**	1.616	−0.349*	−0.165	− 0.127	− 0.022	0.001
(%)	−3.1	−24.0	−267.0	883.5	− 564.8	−27.9	−14.9	29.7	−24.9	0.2
Mother’s height	− 0.058	− 0.051	− 0.005	− 0.079	0.033	8.563***	15.272***	5.641***	4.671***	11.333***
(%)	3.7	3.6	2.2	−79.5	−11.4	686.0	1382.6	− 1316.6	5274.9	1693.1
Mother’s Education	−0.071	0.276	0.058	0.563	0.670	−0.011	−0.325***	− 0.012	0.021	− 0.027
(%)	4.5	−19.3	−25.1	566.5	− 234.2	−0.9	− 29.5	2.8	23.5	−4.0
Working mother	−1.161	−1.384	−0.530	− 0.326	0.338	0.360	1.104***	0.195*	0.088	0.321**
(%)	73.8	97.0	230.5	− 328.3	−118.1	28.9	99.9	−45.4	99.8	48.0
Empowerment	−0.002	0.057	0.073	0.149	0.217	0.012	−0.141***	− 0.012	− 0.010	− 0.010
(%)	0.1	−4.0	−32.0	149.9	−76.0	1.0	−12.7	2.9	−11.7	− 1.5
Media exposure	−0.196	0.244	0.544	0.288	1.442	0.168	0.241**	0.238***	0.236***	0.324***
(%)	12.5	−17.1	−236.9	290.3	− 503.8	13.4	21.9	−55.5	266.4	48.3
**Religion**										
Hindu										
Muslim/Others	0.028	−0.007	− 0.132*	− 0.175	− 0.318	0.007	− 0.080**	0.078***	0.091***	−0.053
(%)	−1.8	0.5	57.5	−175.7	111.0	0.5	−7.3	− 18.2	103.1	− 7.9
**Caste**										
SC										
ST	0.005	−0.020	0.014	−0.045	0.018	0.017	− 0.007	− 0.007	− 0.011	−0.012
(%)	− 0.3	1.4	−6.1	−45.1	−6.1	1.3	−0.6	1.5	−12.2	−1.8
Others	0.115	0.068	0.159	0.130	−0.240	0.301	−0.674***	0.195	−0.073	0.251
(%)	−7.3	−4.7	−69.2	130.8	84.0	24.1	−61.0	−45.4	−82.9	37.5
**Wealth index**	−0.035	− 0.340	−0.1925	0.032	0.189	−0.426**	−1.095***	− 0.450***	− 0.380***	−0.167
(%)	2.2	23.9	83.7	32.1	−66.0	−34.1	−99.1	105.0	− 429.1	−24.9
**Place of residence**										
Urban										
Rural	0.220	0.314	0.478	−0.058	−0.610	0.214	0.163	0.192	0.284	0.232
(%)	−14.0	−22.0	− 207.8	−58.9	213.0	17.2	14.8	−44.8	320.4	34.7
Constant						−6.524*	−17.687***	−6.443***	− 4.971***	− 11.046***
Residuals	1.189	1.315	0.623	0.581	1.278	−1.836	−2.009	−0.889	−1.209	− 1.340
Total	−1.573	− 1.427	− 0.230	0.099	−0.286	1.248***	1.105***	−0.428***	0.089	0.669***

****p* < 0.001; ***p* < 0.01; **p* < 0.05; Source: Computed by the authors from unit-level data of NFHS 2 and NFHS 3

**Table 11:** Oaxaca Blinder decomposition of HAZ scores of NFHS 3 and NFHS 4 in Bihar

	Covariate effect					Coefficient effect				
	10	25	50	75	90	10	25	50	75	90
Aggregate effect										
Child age in months	−0.106***	− 0.263***	−1.026***	− 0.509***	− 0.608***	− 0.781**	− 0.069	3.122***	− 0.519	1.593***
(%)	−35.2	25.4	40.1	−11.9	− 69.5	− 5236.4	−20.7	230.8	41.6	−530.1
Age2	0.022	0.111*	0.503***	0.388***	0.493***	0.551**	0.195	−0.537	0.075	− 0.973***
(%)	7.3	−10.7	−19.7	9.1	56.4	3694.6	58.8	−39.7	−6.0	324.0
*Female*	− 0.001	− 0.005	− 0.026	0.008	− 0.006	0.124***	0.096	− 0.314***	0.133*	0.001
(%)	−0.4	0.5	1.0	0.2	−0.7	829.3	29.1	−23.2	− 10.7	−0.4
**Birth Size**										
*Normal*										
*Average and above*	0.006	−0.028*	− 0.009	− 0.009	− 0.040**	0.014	0.074**	0.043	0.074**	0.100**
(%)	2.0	2.7	0.4	−0.2	−4.6	96.6	22.4	3.2	−6.0	−33.3
*Small*	−0.038***	−0.118***	− 0.195***	− 0.052***	0.041**	0.011	0.112***	0.195***	0.084***	0.003
(%)	−12.7	11.3	7.6	−1.2	4.7	72.5	33.8	14.4	−6.8	− 1.1
Early Breastfeeding (Yes)	0.231**	0.109	−0.167	0.615***	0.068	0.192**	0.022	−0.069	0.411***	0.069
(%)	76.7	−10.5	6.5	14.4	7.7	1287.5	6.7	−5.1	−33.0	−23.1
No. of Siblings	−0.071***	−0.069***	− 0.146***	−0.013	− 0.030*	0.066	0.179**	0.530***	−0.001	0.034
(%)	−23.6	6.7	5.7	−0.3	−3.4	441.6	54.0	39.2	0.1	−11.5
Benefitted ICDS services	0.381***	0.483**	0.653	0.660***	1.121***	0.252*	0.239	0.374	0.392**	0.522**
(%)	126.4	−46.5	−25.5	15.4	128.2	1687.2	72.0	27.6	−31.4	−173.6
**Mother’s Characteristics**										
*Institutional Delivery (yes)*	−0.034	− 0.802***	−1.317***	0.392***	0.086	0.062	−0.694***	−1.364***	0.253	0.082
(%)	−11.4	77.3	51.4	9.2	9.8	415.8	−209.5	−100.8	−20.3	−27.3
Age of mother at first birth	0.152***	−0.013	− 0.077	0.295***	−0.009	1.340***	−0.136	− 0.607	2.734***	1.166
(%)	50.3	1.2	3.0	6.9	−1.0	8980.2	−40.9	−44.9	− 219.3	−388.1
BMI of mother	0.021	−0.068**	− 0.311***	− 0.080***	−0.033	1.470**	0.219	−3.125***	−0.435	0.173
(%)	7.1	6.6	12.1	−1.9	−3.8	9850.3	65.9	−231.0	34.9	−57.5
**Mother’s anaemia**										
Yes	−0.020**	0.044***	0.184***	−0.050***	− 0.030*	− 0.006	− 0.169*	−0.653***	0.195*	0.091
(%)	−6.8	−4.3	−7.2	−1.2	−3.4	−37.6	−51.0	− 48.3	− 15.7	− 30.2
Mother’s height	− 0.006	− 0.012	− 0.027	−0.003	− 0.008	2.556	−2.430	−12.737***	7.506***	0.629
(%)	−1.8	1.2	1.0	−0.1	− 0.9	17,128.9	− 732.9	− 941.6	− 601.9	− 209.3
Mother’s Education	0.099***	0.124***	0.227***	0.100***	−0.101***	0.229***	0.265***	0.417***	0.004	−0.141
(%)	33.0	−12.0	−8.9	2.3	−11.5	1533.2	79.9	30.8	−0.3	47.1
Working mother	0.089***	0.128***	− 0.026	0.019	−0.008	0.382*	0.554**	−0.260	−0.013	0.157
(%)	29.6	−12.3	1.0	0.4	−0.9	2558.2	167.1	−19.2	1.1	−52.2
Empowerment	0.018	−0.008	0.041	0.057***	−0.070***	− 0.001	0.001	− 0.003	− 0.006	0.002
(%)	6.0	0.8	−1.6	1.3	−8.0	− 8.7	0.2	−0.2	0.5	−0.6
Media exposure	0.137**	0.144	−0.013	0.310***	0.088	0.092	0.114	−0.006	0.334***	0.084
(%)	45.3	−13.9	0.5	7.2	10.1	618.9	34.3	−0.4	−26.8	−28.0
**Religion**										
Hindu										
Muslim/Others	−0.018*	0.011	0.019	−0.055**	− 0.035*	0.115**	−0.082	− 0.128	0.245***	0.125*
(%)	−5.9	−1.0	−0.7	−1.3	−4.0	770.7	− 24.7	−9.5	−19.7	−41.6
**Caste**										
SC										
ST	−2.036***	−1.713***	−3.193***	0.831**	0.058	−1.150***	−1.044***	− 1.970***	0.470*	0.002
(%)	−674.7	165.1	124.7	19.4	6.7	− 7705.5	− 315.1	− 145.6	− 37.7	− 0.7
Others	1.510***	0.968	2.434	1.425**	−0.138	−0.256***	− 0.159	−0.414	− 0.254**	0.012
(%)	500.6	−93.3	−95.1	33.3	−15.8	− 1714.8	−48.1	−30.6	20.4	− 4.1
**Wealth index**	−0.039**	−0.055**	− 0.110**	−0.056***	0.020	0.355***	0.328**	0.801**	0.271*	−0.155
(%)	−12.9	5.3	4.3	−1.3	2.3	2381.6	98.9	59.2	−21.7	51.7
**Place of residence**										
Urban										
Rural	0.004	−0.005	0.020	0.008	0.014	0.076	− 0.246	0.906**	0.163	0.816***
(%)	1.2	0.5	−0.8	0.2	1.6	510.8	−74.3	66.9	−13.1	− 271.5
Constant						−5.678**	2.965	17.154**	− 13.367***	−4.692
Residuals	− 0.507	1.099	2.728	− 4.015	−0.846	− 0.258	− 0.754	−1.831	0.601	− 0.262
Total	0.302	−1.037	−2.560	4.281***	0.874	0.015	0.332	1.353	−1.247***	−0.300

****p* < 0.001; ***p* < 0.01; **p* < 0.05; Source: Computed by the authors from unit-level data of NFHS 3 and NFHS 4

## Abbreviations

NFHS: National Family Health Survey
DHS: Demographic Health Survey
AOR: Adjusted Odds Ratio
RIF: Re-Centred Influence Function
HAZ: Height for age Z score
SDG: Sustainable Development Goals
IIPS: International Institute for Population Sciences

## Footnotes

1 Women’s empowerment indicators were created from factor scores of the factor analyses using different variables indicating women’s household decision making power, freedom of movement etc. For NFHS 2, 1998-99, the following variables were included: who decides how to spend money, who decides about obtaining health care, who decides what to cook, permission needed to go to market, and permission needed to visit relatives or friends. Women’s work for cash in the past 12 months was also incorporated. In NFHS 3, 2005-06, final say on how to spend money, final say on one’s own health care, final say on household purchases, final say on visiting relatives or friends, work for cash in the past 12 months, having bank account were considered to create such index. In NFHS 4, 2015-16, the variables such as who decides on own health care, who decides on how to spend money, who decides on household purchases, who decides about visiting relatives or friends, owning house/land, work for cash in the past 12 months, having bank account, and having mobile phone were included in the analysis.

2 JEEViKA is an initiative of the Government of Bihar for poverty alleviation, which aims at social and economic empowerment of the rural poor by improving their livelihoods and by developing institutions of women like self-help groups (SHGs) and their federations. It will eventually enable rural households to access and negotiate better public provisioning of credit, assets and services [50]
